# Stratification of Estrogen Receptor-Negative Breast Cancer Patients by Integrating the Somatic Mutations and Transcriptomic Data

**DOI:** 10.3389/fgene.2021.610087

**Published:** 2021-02-03

**Authors:** Jie Hou, Xiufen Ye, Yixing Wang, Chuanlong Li

**Affiliations:** College of Intelligent Systems Science and Engineering, Harbin Engineering University, Harbin, China

**Keywords:** breast cancer patient stratification, estrogen receptor-negative, distance correlation, significantly mutated gene, gene coexpression network

## Abstract

Patients with estrogen receptor-negative breast cancer generally have a worse prognosis than estrogen receptor-positive patients. Nevertheless, a significant proportion of the estrogen receptor-negative cases have favorable outcomes. Identifying patients with a good prognosis, however, remains difficult, as recent studies are quite limited. The identification of molecular biomarkers is needed to better stratify patients. The significantly mutated genes may be potentially used as biomarkers to identify the subtype and to predict outcomes. To identify the biomarkers of receptor-negative breast cancer among the significantly mutated genes, we developed a workflow to screen significantly mutated genes associated with the estrogen receptor in breast cancer by a gene coexpression module. The similarity matrix was calculated with distance correlation to obtain gene modules through a weighted gene coexpression network analysis. The modules highly associated with the estrogen receptor, called important modules, were enriched for breast cancer-related pathways or disease. To screen significantly mutated genes, a new gene list was obtained through the overlap of the important module genes and the significantly mutated genes. The genes on this list can be used as biomarkers to predict survival of estrogen receptor-negative breast cancer patients. Furthermore, we selected six hub significantly mutated genes in the gene list which were also able to separate these patients. Our method provides a new and alternative method for integrating somatic gene mutations and expression data for patient stratification of estrogen receptor-negative breast cancers.

## 1. Introduction

Breast cancer is a heterogeneous disease with many subtypes which exhibits significant differences in response to therapy and patient outcomes (Jonasson et al., 2019). Breast cancer has been known to be an endocrine-related cancer (Wu et al., 2020), and the majority of breast cancer subtypes are hormone-associated (DeSantis et al., 2017; Xu et al., 2019). The expression of the estrogen receptor (ER), progesterone receptor (PR), and human epithelial growth factor receptor 2 (HER2) as predictive and/or prognostic markers has been well established in multiple studies (Francis et al., 2019). Endocrine therapies that target the ER have long been the cornerstone of therapy approaches for the majority of breast cancer patients. However, 20–30% of breast tumors do not express ER and are not responsive to existing endocrine therapies (Ni et al., 2011). The prognosis of estrogen receptor-negative (ER^−^) breast cancer is worse than estrogen receptor-positive (ER^+^) breast cancer in most situations, but ER^−^ breast cancer patients do not always have a poor clinical outcome. Due to the lack of reliable biomarkers, it is impossible to identify ER^−^ tumors with a good prognosis (Teschendorff et al., 2007; Zhang et al., 2016). Several studies have revealed that different chromosomal and gene expression patterns are present in patients with different estrogen receptor statuses (Zhang et al., 2009; Fohlin et al., 2020). Thus, an accurate grouping of ER^−^ breast cancer into clinically relevant subtypes is of particular importance for therapeutic decision making.

Cancer is often driven by the accumulation of genetic alterations. Until now, the somatic mutation landscapes and signatures of more than a dozen major cancer types have been reported. However, pinpointing the driver mutations and cancer genes from millions of available cancer somatic mutations remains a significant challenge (Cheng et al., 2016). In The Cancer Genome Atlas (TCGA) database, a phenomenon can be observed that the position and nature of somatic mutations can often be translated to changes of protein structures or functions of the genes. The affected gene is designated as a significantly mutated gene (SMG). SMGs are the result of splice-site change, nonsense, nonstop, or frame-shift mutations (Zhang et al., 2016). The prevalence of SMGs in almost all cancer types has allowed for postulation that they may be act potentially as biomarkers for subtyping and testing for use in cancer patient outcome predictions, or a starting point of clarifying the tumorigenesis process (Cancer Genome Atlas Network, 2012).

Network approaches have provided the means to bridge the gap between individual genes and systems oncology (Ghazalpour et al., 2006). Weighted gene coexpression network analysis (WGCNA) is a systems biology method used to analyze gene expression profiling data which is widely used in bioinformatics (Zhang and Horvath, 2005). WGCNA can help researchers analyze the relationships between genes and pathogenic mechanisms. Instead of linking thousands of genes to the disease, this method focuses on the relationship between gene modules and disease traits (Huang et al., 2020). Many studies that constructed the coexpression networks in breast cancer used WGCNA. Coexpression networks were used to screen hub genes from the co-expression module using the relationship between genes and traits, together with the core position of genes in the module (Tang et al., 2019; Jia et al., 2020). A coexpression network analysis can also identify the prognostic lncRNAs (Liu et al., 2019; Li et al., 2020). However, these studies did not consider the information of genetic mutations in breast cancer.

SMGs are not always concentrated in specific genomic loci, which suggests that instead of common genes, mutations may affect some pathways or gene interaction networks (Zhang et al., 2016). So, in this work, we propose a method to screen SMGs using gene coexpression networks to identify the SMGs that highly relate to ER_Status. We show the development of a workflow for screening SMGs associated with clinical data of the estrogen receptor in breast cancer by a gene coexpression module. The new gene list was designated as important-SMGs. The identified genes, which were used to stratify patients with different subtypes of cancers, were suggested to represent biomarkers. Our method provides a new alternative method for cancer patient stratification by integrating transcriptomic, variants, and clinic data.

## 2. Methods

**Figure 1:**
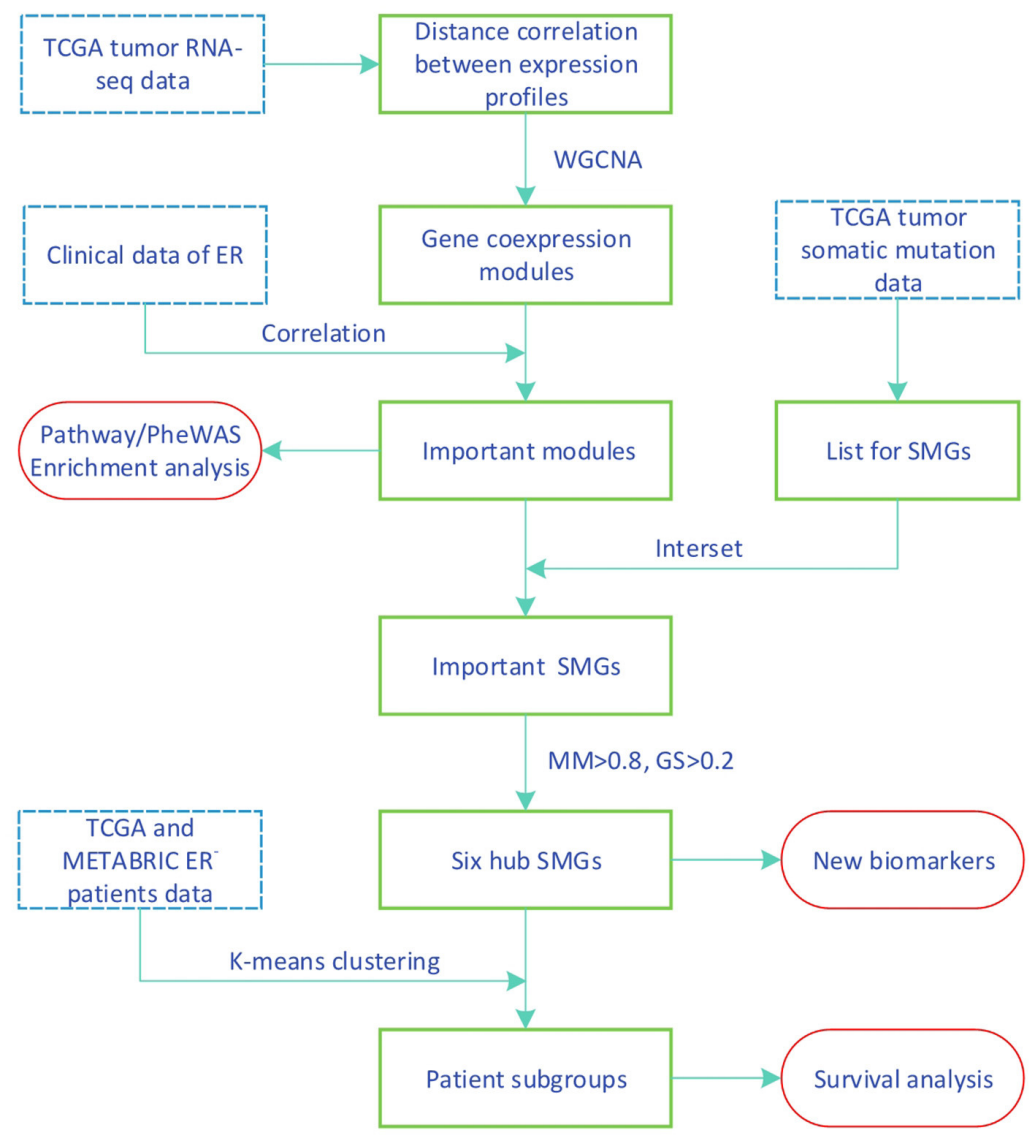
Workflow of identifying new biomarkers using transcriptomic and variants data.

In this work, we propose a method for screening SMGs by a gene coexpression module associated with clinical data of breast cancer and the estrogen receptor; the workflow is summarized in Figure 1. We calculated the similarity coexpression matrix by distance correlation for WGCNA to construct a gene coexpression network and to obtain the gene modules. Distance correlation has a perfect theoretical system and works for both linear and nonlinear dependence between any two vectors (Székely et al., 2007). WGCNA is a method used to identify clusters (modules) of highly correlated genes (Zhang and Horvath, 2005). We identified some important modules that were significantly associated with the measured clinical estrogen receptor data. SMGs were then selected from the TCGA tumor somatic mutation data and the important-SMGs were obtained through the overlap between the important module genes and the SMGs. Furthermore, we respectively chose the hub SMGs in the important modules and acquired six genes which can be used as the biomarkers for stratification and prediction of patient survival of ER^−^ breast cancer.

### 2.1. Datasets

The TCGA datasets used in this study can be found in the Data Portal for TCGA-Breast Cancer (Weinstein et al., 2013), For the construction of the gene coexpression and the SMGs selection, we used the TCGA dataset. The gene expression profile was measured experimentally using the Illumina HiSeq 2000 RNA Sequencing platform with *log*_2_(*x*+1) transformed RSEM normalized count (Cancer Genome Atlas Network, 2012). The samples were screened based on RNA-seq data and clinical data, after which we selected genes with a variable coefficient of more than 0.2 and a mean >1. Ultimately, we obtained 5,076 genes.

The Molecular Taxonomy of Breast Cancer International Consortium (METABRIC) dataset from the cBioportal website (Cerami et al., 2012) contains cDNA microarray performed on the Illumina HT-12 platform (Curtis et al., 2012; Pereira et al., 2016). The details of data normalization can be found in Margolin et al. (2013). For validation, both datasets containing gene expression data and matching survival time (months) were used for survival analysis. Samples in the METABRIC were screened based on the clinical data (contain ER_Status, Days, Vital_Status). The sample numbers used in the two datasets are shown in Table 1.

**Table 1:** Sample numbers in two datsets.

**Dataset**	**Total**	**SMGs**	**ER^+^**	**ER^−^**	**Deceased/Living (ER^−^)**
TCGA	637	383	499	133	23/110 ≈ 0.209
METABRIC	1,888	–	1,435	424	240/184 ≈ 1.304

*There are more samples in METABRIC and longer clinical follow-up time*.

### 2.2. Distance Correlation

In 2007, distance correlation was proposed by Szekely, Rizzo, and Bakirov in the paper titled *Measuring and Testing Dependence by Correlation of Distances* published in the Annals of Statistics (Székely et al., 2007). In this work, the similarity coexpression matrix was calculated with distance correlation for WGCNA to perform a gene coexpression network analysis. Unlike the Pearson correlation, distance correlation works for both linear and nonlinear dependence between two gene expression profiles. However, distance correlation is still a relatively expensive computation. The time complexity of distance correlation was *O*(*n*^2^). Distance correlation was calculated using the energy package in R (see the references in the manual for more package details).

### 2.3. WGCNA

WGCNA (Zhang and Horvath, 2005) can be used to identify clusters (modules) of highly correlated genes. This method summarizes such clusters using the module eigengene or an intramodular hub gene. Alternatively, it relates modules to one another and to external sample traits and calculating module membership measures using the eigengene network methodology (Langfelder and Horvath, 2008; Luo et al., 2018). The functions of WGCNA are plentiful, and only some of them were used in this study. We mainly used the process of module division of WGCNA. First, the correlation for all genes was calculated using correlation methods, and a similarity coexpression matrix was obtained. The similarity coexpression matrix was transformed to an adjacency matrix using the soft-thresholding power which was chosen based on the criteria of approximating the scale-free topology (SFT) of the network. Next, a topological overlap matrix was computed from the adjacency matrix. Finally, a tree (dendrogram) was produced from the dissimilarity topological overlap matrix by hierarchical clustering. The clusters (modules) were obtained using dynamic tree cutting. For functions of WGCNA, we refer to the corresponding tutorials package. The WGCNA package is now available from the *Comprehensive R Archive Network*(CRAN).

### 2.4. Enrichment Analysis

Enrichr (Chen et al., 2013; Kuleshov et al., 2016) was used to analyze the Kyoto Encyclopedia of Genes and Genomes (KEGG) (Kanehisa et al., 2019) pathways and the phenome-wide association studies (PheWAS) (Denny et al., 2010) of diseases identified in the important modules. Enrichr is open source and freely available online.

### 2.5. SMGs and Important SMGs

The SMGs were obtained by screening the somatic mutations derived from the TCGA breast cancer patients. The SMGs are genes with frame-shift indels, splice-site changes, nonstop mutations, or nonsense mutations (Zhang et al., 2016). Mismatch, silent, RNA, and in-frame indel mutations did not belong to the SMGs. Among the samples we selected, the mutation types of 1920 SMGs and 383 samples are listed in Supplementary Table 1.

To obtain ER-related SMG, we acquired some SMGs contained in the important modules by taking the intersection of genes in important modules and SMGs, and we named them important SMGs.

### 2.6. Gene Significance and Module Membership

To find genes associated with clinical ER_Status, we defined a measure of gene significance (GS) between the *i*-th gene profile *x*_*i*_ and the ER_Status as

(1) $$\begin{array}{r} {\text{GS}}_{i}=cor\left(x_{i},\text{ER\_Status}\right), \end{array}$$

where *cor*(·, ·) denotes the correlation coefficients. ER_Status can be mapped to a binary indicator variable where 1 is positive and 0 is negative. The higher the absolute value of GS_*i*_ of the gene, the more closely relevant it is to ER.

To measure the relationship between the *i*-th gene and the module to which it belongs, we introduced the module membership (MM) (Langfelder and Horvath, 2008; Wei et al., 2020) which was defined by calculating the correlation coefficient between the gene expression profile and the module eigengene.

### 2.7. Survival Analysis

**Figure 2:**
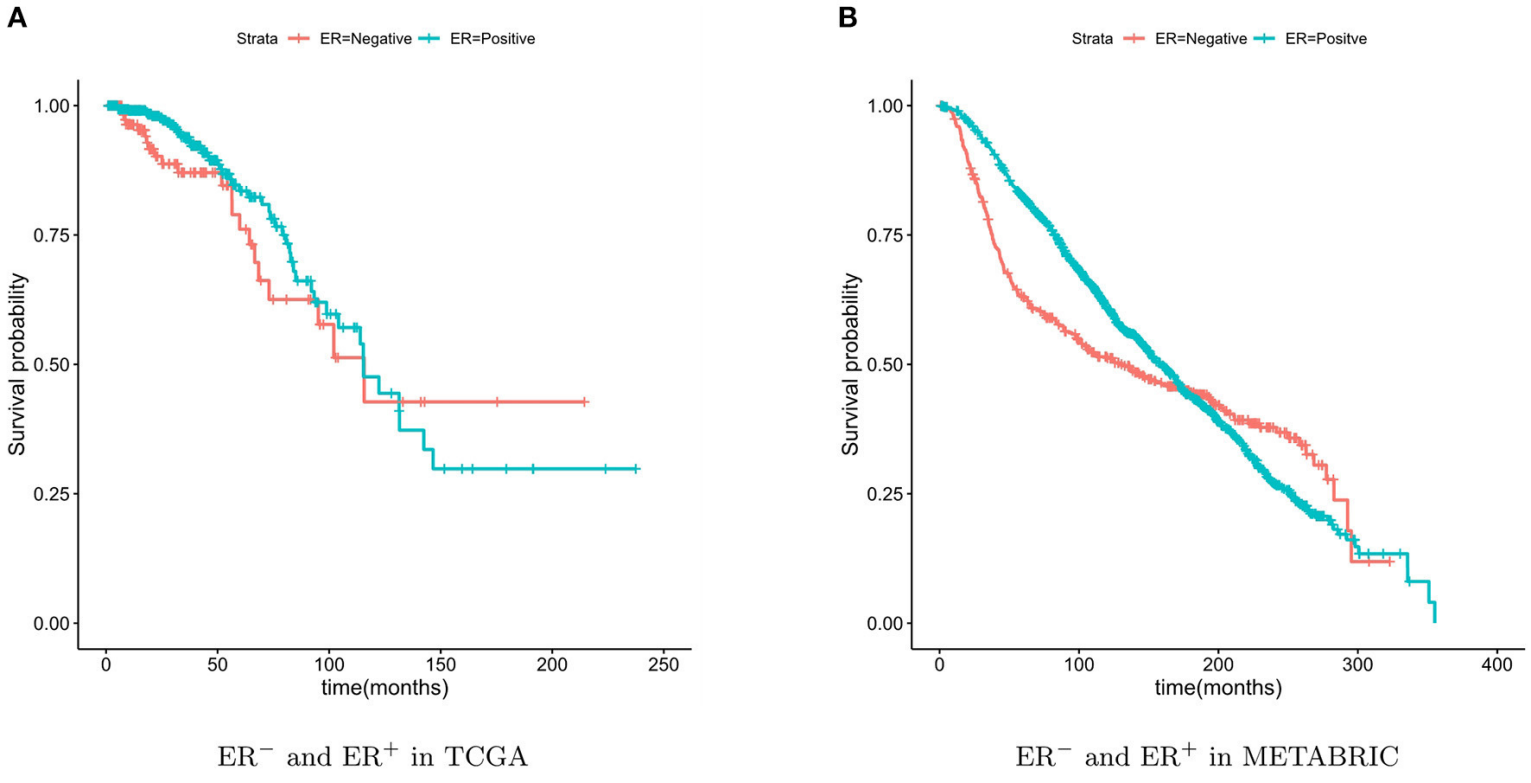
Kaplan-Meier survival curves of ER^−^ and ER^+^. The ER^−^ breast cancer patient have a poor prognosis in the short term and a relatively good prognosis in the longer term.

Some subtypes of breast cancer have a poor prognosis in the short term and a relatively good prognosis in the longer term. This particular characteristic of ER^−^ breast cancer can be observed from Figure 2. Due to this characteristic, the two survival curves may cross. This made the log-rank test *P*-value large, although the two curves were obviously separate. The two-stage hypothesis test was developed for handling the crossing hazard rates problem. We evaluated the *P*-values of both the log-rank and the two-stage hypothesis tests.

For validation, the TCGA breast cancer dataset (containing 133 ER^−^ patients) and the METABRIC dataset (containing 424 ER^−^ patients) were used. The breast cancer characteristic led to the crossing of the two survival curves, so the two-stage hypothesis test was developed for handling the crossing hazard rates problem (Qiu and Sheng, 2008). To predicate the significance of the difference in the survival time between the two patient groups, we performed the Log-rank and two-stage tests.

## 3. Results

### 3.1. Gene Co-expression Module Associated With Estrogen Receptor

**Figure 3:**
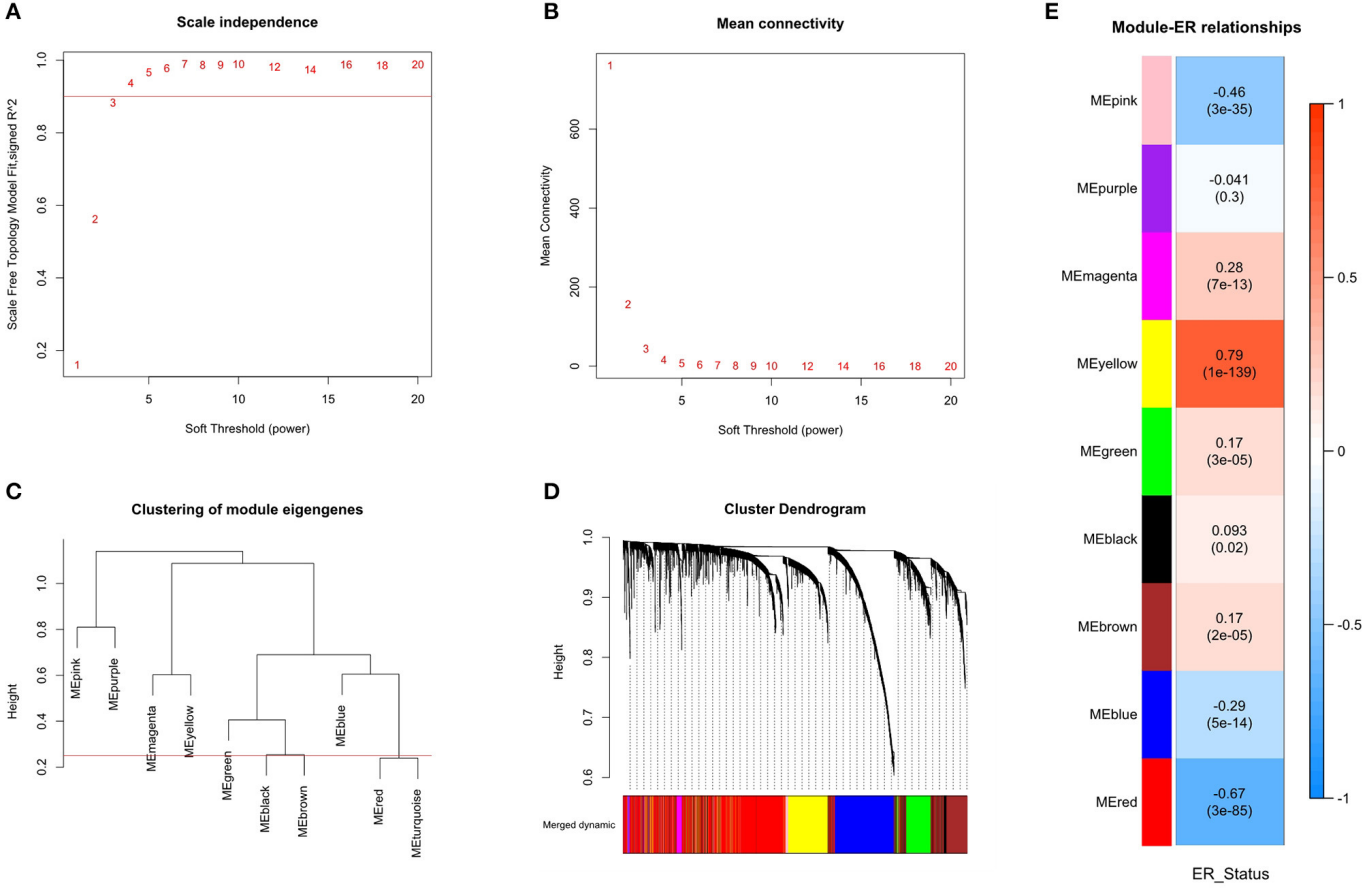
Identification of modules associated with the ER_Status of breast cancer. **(A)** The scale-free fit index for various soft-thresholding power. Scale-free topology (SFT) was achieved when the recommended soft-thresholding power was 3. **(B)** The mean connectivity for various soft-thresholding powers. **(C)** The cluster dendrogram of module eigengenes. **(D)** The cluster dendrogram of all genes with corresponding color assignments. Nine colors present nine modules. **(E)** Module-ER_Status relationship heatmap. The values above the brackets represent the correlation coefficients between modules and ER_Status. The values in brackets are the *P*-values for the association test. The red and yellow modules were significantly related to the ER_Status and selected as the important modules.

The similarity coexpression matrix was calculated with distance correlation. When we chose 3 as the recommended soft-thresholding power, the SFT was achieved. The scale-free fit index is shown in Figure 3A, and the mean connectivity for various soft-thresholding powers is shown in Figure 3B. The modules were obtained by hierarchical clustering based on the minimum module size of 30. The modules were then merged if the similarity between module eigengenes were >0.75. The cluster trees (dendrograms) of the module eigengenes are shown in Figure 3C and the cluster dendrograms of the genes that were assigned module colors after the merge is shown in the Figure 3D. Finally, nine coexpression modules were constructed.

To find modules related to clinical ER_Status, the correlation between modules eigengenes and ER_Status was calculated and shown in Figure 3E. The modules eigengenes were associated with ER_Status when *p* < 0.01. There were four modules positively associated with ER_Status, and three modules that were negatively associated. The yellow and red modules, where the absolute value of the correlation coefficient was >0.6, had the highest correlations with ER_Status. This means that these modules have great biological significance related to the ER_Status, so these two modules were selected as the important modules.

### 3.2. Enrichment Analysis of the Important Modules

We analyzed the KEGG and PheWAS enrichments for the two important modules to associate each module with biological pathways and diseases (see Table 2). Enrichment results of all modules are available in Supplementary Table 2.

**Table 2:** KEGG and PheWAS enrichment analysis by Enrichr of the important modules identified by WGCNA.

**Module**	**No**.	**KEGG**	***P*-value**	**PheWeb**	***P*-value**
Yellow	677	Dilated cardiomyopathy (DCM)	3.67E-03	Cancer of stomach	2.18E-03
		Adrenergic signaling in cardiomyocytes	3.86E-03	Pelvic peritoneal adhesions,-	5.20E-03
				female (postoperative) (postinfection)	
		Cardiac muscle contraction	4.83E-03	Cholecystitis without cholelithiasis	5.85E-03
		Glutamatergic synapse	5.35E-03	Cancer of eye	8.57E-03
		Hypertrophic cardiomyopathy (HCM)	8.07E-03	Elevated cancer antigen 125 [CA 125]	8.57E-03
Red	1819	Metabolism of xenobiotics-	2.80E-04	Genital prolapse	6.45E-04
		by cytochrome P450			
		Chemical carcinogenesis	3.42E-04	Breast cancer	2.55E-03
		Neuroactive ligand-receptor interaction	1.31E-03	Osteoarthrosis, localized, primary	2.73E-03
		Caffeine metabolism	6.53E-03	Heart failure with preserved	2.88E-03
				EF [Diastolic heart failure]	
		Protein digestion and absorption	6.83E-03	Other venous embolism and thrombosis	4.09E-03

*All the important modules were highly enriched with PheWAS in breast cancer, cancer or female-related diseases*.

Several KEGG enriched terms related to cardiac diseases were enriched in the yellow module. Approximately 59% of cancer patients in the dataset used in this study received radiation therapy. What is more, hormonal therapy plays an important role in breast cancer treatments (Jones and Buzdar, 2004). Some reports showed that one of the side effects of breast cancer treatments (radiation therapy, hormonal therapy) is cardiotoxicity (Bird and Swain, 2008; Demissei et al., 2020). This may be the cause of the enrichment of the cardiac disease pathway in the yellow module. The yellow modules were highly enriched in cancer (For instance, cancer of stomach, cancer of eye, and elevated cancer antigen) or female-related diseases with PheWAS. With the KEGG pathway enrichment analysis, the red modules were enriched in the metabolism and chemical carcinogenesis pathways. This is consistent with the conclusion that the ER is a modulator in metabolic disorders (Mauvais-Jarvis et al., 2013). With PheWAS diseases enrichment analysis, the top two significant terms were breast cancer and female-related diseases. The results of the enrichment analysis confirmed the biological significance of the important modules related to breast cancer or other cancers.

### 3.3. Survival Analysis by Important-SMGs and RNA-Seq Data

The new gene list, designated as the important-SMGs, was obtained through overlapping the important module genes and the SMGs. The list contains 227 SMGs and is shown in Supplementary Table 3.

**Figure 4:**
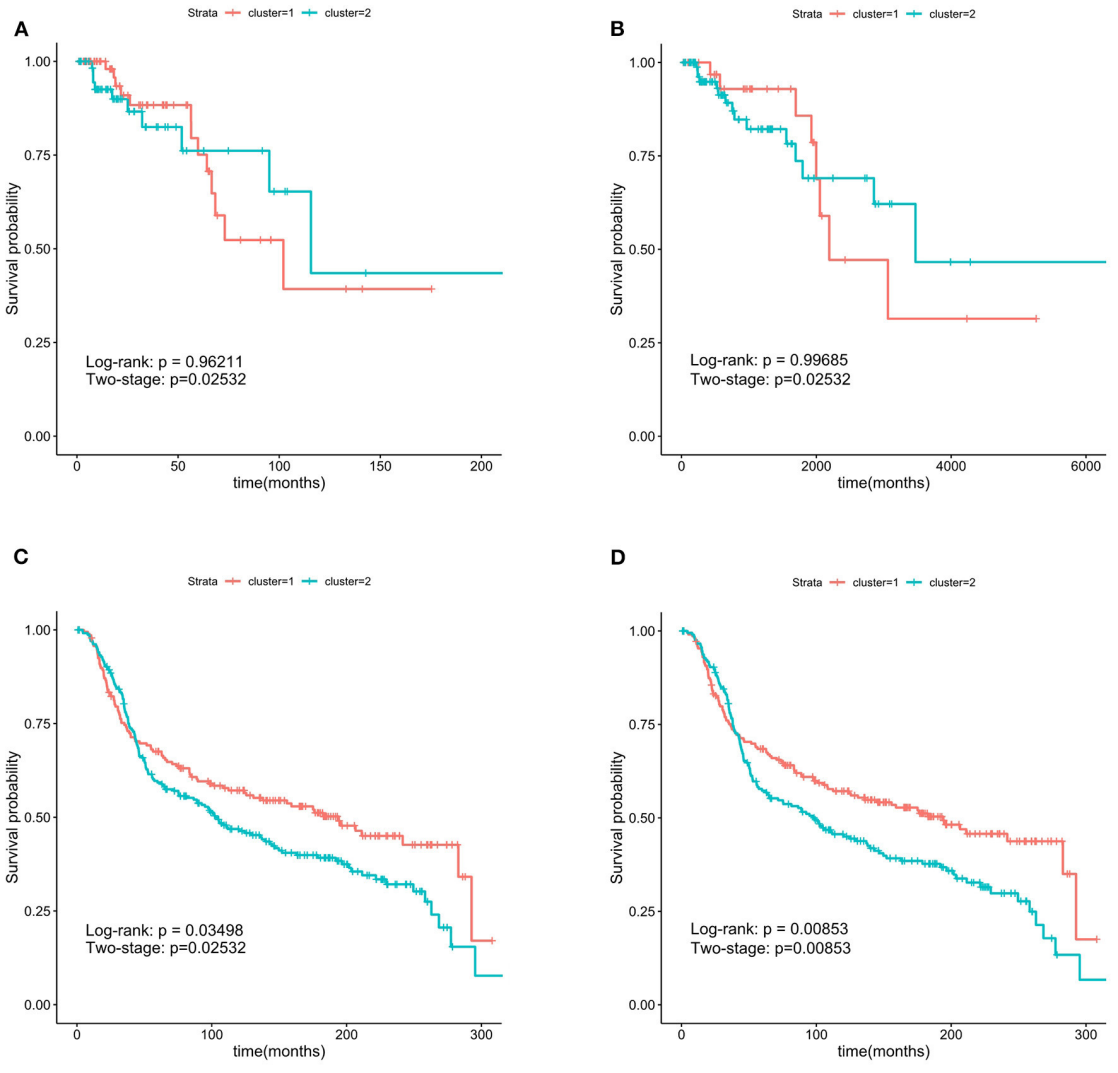
Kaplan-Meier survival curves. The 227 important-SMGs were able to separate the patients into two groups more significantly. The *P*-values were smaller in METABRIC dataset. **(A)** Group 1 in TCGA, **(B)** important-SMGs in TCGA, **(C)** group 1 METABRIC, **(D)** important-SMGs in METABRIC.

In Zhang et al. (2016), the ER^−^ samples were also separated into two groups. The authors developed an approach for stratifying cancer patients into groups with different clinical outcomes. They focused on this specific Group 1 with a significantly higher proportion of ER-negative patients. Thirteen SMGs among the 201 SMGs in Group 1 are identical to the important-SMGs obtained by our approach. The TCGA breast cancer dataset (containing 133 ER^−^ patients) and the METABRIC dataset (containing 424 ER^−^ patients) were used in this test. The important-SMGs in this work were compared with the Group 1-specific genes in Zhang et al. (2016). For survival analysis, the ER^−^ samples were separated into two groups based on the K-means algorithm with K = 2, using the two gene lists and the RNA-seq data. The results are shown in Figure 4.

From the two-stage *P*-value, the two gene lists in our test on the TCGA ER^−^ data were able to separate the patients into two groups with a significant survival time difference. The survival curves in Figures 4A,B were clearly separated, but the two curves obtained by the important-SMGs in Figure 4B were further apart than that obtained by the gene list of Group 1 in Zhang et al. (2016) in Figure 4A. Therefore, on the TCGA ER^−^ data, the important-SMGs were able to separate the patients into two more significant groups.

The test on METABRIC data shown in Figure 4D suggested that the important-SMGs were able to separate the patients into two groups with a significant survival time difference (the *P*-value of the two tests are 0.00853). However, the gene list of Group 1 in Zhang et al. (2016) shown in Figure 4C could effectively separate the ER^−^ patients with the bigger *P*-value (the *P*-values of the two tests larger than 0.01). The survival curves of the two groups obtained by the important-SMGs were also further apart. Therefore, on the METABRIC data, the important-SMGs were able to separate the patients into two more significant groups.

### 3.4. Survival Analysis by Six Hub SMGs and RNA-Seq Data

As discussed in the previous section, the 227 important-SMGs were able to more significantly separate the ER^−^ patients into two groups. As biomarkers, it is best to keep the number of genes as small as possible. Gene co-expression modules were composed of highly correlated genes, we just have to choose a few representative genes from 227 SMGs. The most representative genes are the hub genes within important modules.

**Figure 5:**
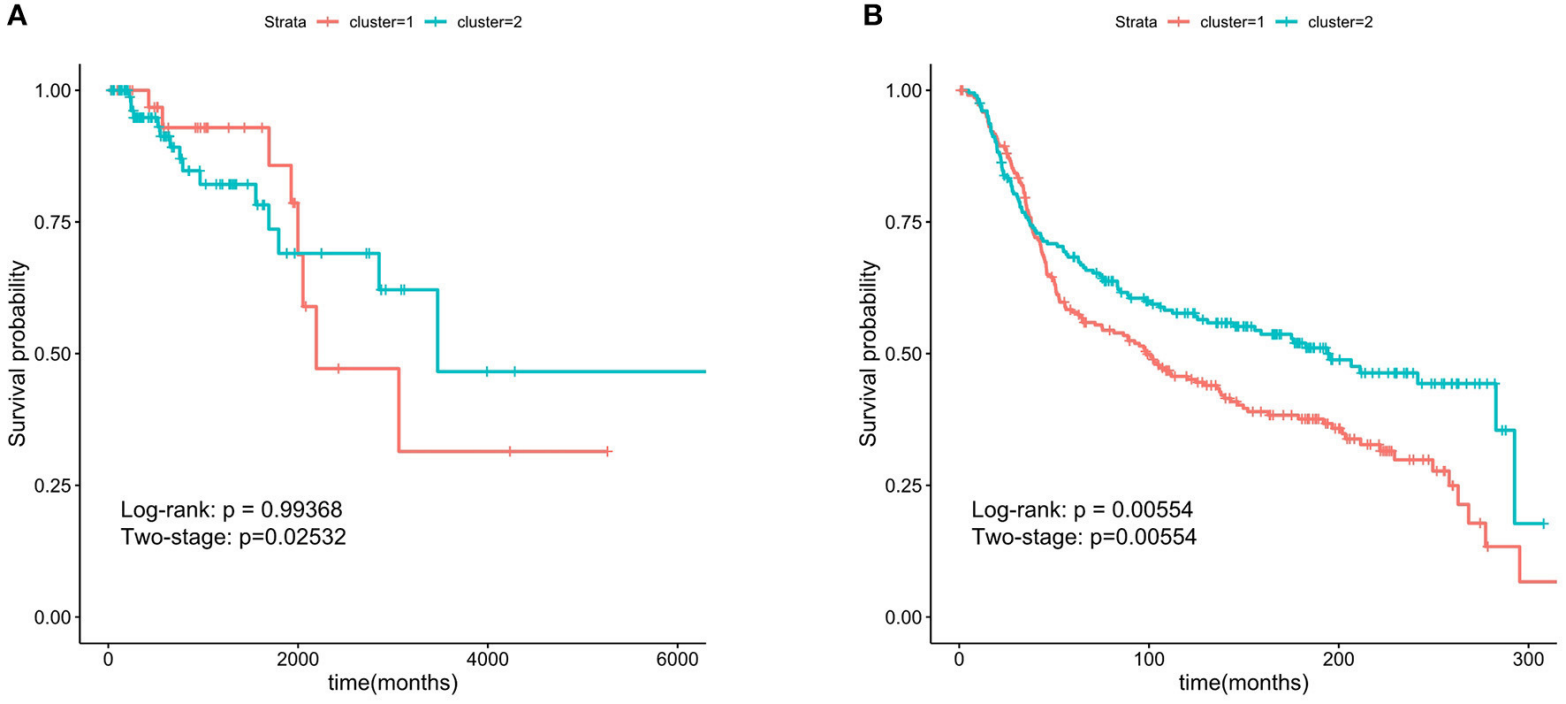
Kaplan-Meier survival curves using the six hub SMGs. A few hub SMGs can represent the 227 important-SMGs and were able to separate the patients into two groups more significantly. **(A)** Six hub SMGs in TCGA. **(B)** Six hub SMGs in METABRIC.

We chose the GS>0.2 and MM>0.8 in the two important modules and obtain 29 hub genes. The six genes (FOXA1, GABRP, BCL11A, DNALI1, STAC, and ESR1) obtained by overlapping the 29 hub genes and the SMGs were called the hub-SMGs. The ER^−^ samples were separated into two groups based on the K-means algorithm with *K* = 2, using the hub-SMGs and the RNA-seq data. The results in the TCGA and the METABRIC datasets of survival analysis are shown in Figure 5. From the value of the two-stage *P*-value, the hub-SMGs can significantly separate the ER^−^ breast cancer patients into two groups. Patients in different groups have different survival times. From Figure 5B, the P-value in the METABRIC dataset is 0.00554 which is smaller than the *P*-value of the Important-SMGs 0.00853 (see Figure 4D). This suggests that a few genes can represent the important-SMGs and separate the ER^−^ patients.

## 4. Conclusion

With rapid developments in massively parallel sequencing and computing capacity, a rich resource of data in different modalities for cancer specimens have been generated in public databases at an amazing speed. Therefore, integrating and mining the tremendous volume of data has become an important subject in the field of bioinformatics. In our study, we show the development of a new workflow to integrate somatic mutations, gene expression, and clinical data. We constructed a gene co-expression network and obtained nine coexpression modules. The yellow and red modules were selected as the important modules, because these two modules have the most significant correlation with ER. We obtained the important-SMGs list through the overlap between the important module genes and the SMGs. In the TCGA and METABRIC datasets, we verified that the important-SMGs were able to separate the ER^−^ patients more significantly than other methods.

Furthermore, we selected the six hub SMGs as potential biomarkers which are also able to separate these patients. The genes ESR1, DNALI1, and FOXA1 belong to the yellow module, the genes GABRP, STAC, and BCL11A belong to the red module. These six genes have been reported to be related to cancer or breast cancer in the literature. In particular, two genes in the yellow module are directly related to estrogen receptors. ESR1 (estrogen receptor 1, also known as ER) is a gene that encodes the estrogen receptor protein (Holst et al., 2007). FOXA1 is a key determinant of estrogen receptor function and endocrine response (Hurtado et al., 2011). The conclusion of the relevant literature verified the correctness of our algorithm flow.

Our work provided a novel workflow for identifying new biomarkers using transcriptomic and variants data. In future research, we will use the same workflow for other complex diseases to further test its effectiveness and to find a new gene list to stratify patients.

## Data Availability Statement

Publicly available datasets were analyzed in this study. This data can be found at: https://portal.gdc.cancer.gov/, https://www.cbioportal.org/.

## Author Contributions

XY supervised this work, made critical revisions, and approved final version. JH designed the study, analyzed the data, and wrote the original draft of the manuscript. YW and CL analyzed the data and revised the manuscript. All authors read and approved the final manuscript.

## Conflict of Interest

The authors declare that the research was conducted in the absence of any commercial or financial relationships that could be construed as a potential conflict of interest.
