# Supplementary material for: Stratification of Estrogen Receptor-Negative Breast Cancer Patients by Integrating the Somatic Mutations and Transcriptomic Data
Source: Front Genet. 2021 Feb 3;12:610087. doi: 10.3389/fgene.2021.610087 (PMC7886807; doi:10.3389/fgene.2021.610087)
Supplement: Supplementary file 2 [file Table_2.DOCX]

Table S2 The results for KEGG pathway and PheWeb diseases enrichment analysis (top 5 in each module are listed).

| **Module** | **No.** | **KEGG** | **PValue** | **PheWeb** | **PValue** |
| --- | --- | --- | --- | --- | --- |
| black | 171 | Amphetamine addiction | 2.91E-04 | Chronic kidney disease, Stage I or II | 6.66E-05 |
|  |  | Neuroactive ligand-receptor interaction | 6.80E-04 | Chondrocalcinosis | 5.38E-04 |
|  |  | Dopaminergic synapse | 9.26E-04 | Crystal arthropathies | 5.38E-04 |
|  |  | Neurotrophin signaling pathway | 3.59E-03 | Dental caries | 1.23E-03 |
|  |  | Nicotine addiction | 4.81E-03 | Neoplasm of uncertain behavior of skin | 1.72E-03 |
| blue | 912 | Cytokine-cytokine receptor interaction | 2.61E-36 | Hypothyroidism | 4.84E-03 |
|  |  | Hematopoietic cell lineage | 1.21E-22 | Schmorl's nodes | 8.91E-03 |
|  |  | Chemokine signaling pathway | 9.65E-15 | Pleurisy; pleural effusion | 1.16E-02 |
|  |  | Graft-versus-host disease | 1.08E-11 | Other nondiabetic retinopathy | 2.20E-02 |
|  |  | Inflammatory bowel disease (IBD) | 4.65E-11 | Adrenal cortical steroids causing adverse effects in therapeutic use | 2.36E-02 |
| brown | 828 | Adrenergic signaling in cardiomyocytes | 8.81E-06 | Cerebral ischemia | 2.72E-04 |
|  |  | PPAR signaling pathway | 9.64E-06 | Other disorders of ear | 4.45E-04 |
|  |  | Neuroactive ligand-receptor interaction | 3.17E-05 | Other acquired deformities of limbs | 8.52E-04 |
|  |  | Tyrosine metabolism | 8.99E-05 | Back pain | 1.11E-03 |
|  |  | Dilated cardiomyopathy (DCM) | 3.66E-04 | Erectile dysfunction [ED] | 1.41E-03 |
| green | 452 | Neuroactive ligand-receptor interaction | 3.05E-05 | Corns and callosities | 4.10E-16 |
|  |  | Serotonergic synapse | 1.05E-03 | Cardiac pacemaker in situ | 3.83E-05 |
|  |  | TGF-beta signaling pathway | 4.24E-03 | Peripheral or central vertigo | 3.89E-05 |
|  |  | Long-term depression | 1.13E-02 | Polyp of female genital organs | 1.37E-04 |
|  |  | Renin-angiotensin system | 1.44E-02 | Conduct disorders | 1.55E-04 |
| magenta | 70 | Huntington disease | 3.02E-02 | Systemic sclerosis | 3.45E-02 |
|  |  |  |  | Congenital anomalies of the eye | 3.45E-02 |
|  |  |  |  | Encephalitis | 3.45E-02 |
|  |  |  |  | Balanoposthitis | 3.45E-02 |
|  |  |  |  | Systemic sclerosis | 3.45E-02 |
| pink | 105 | Small cell lung cancer | 1.30E-02 | Postlaminectomy syndrome | 3.12E-03 |
|  |  | Purine metabolism | 3.05E-02 | Fracture of radius and ulna | 7.57E-03 |
|  |  | Gastric cancer | 4.38E-02 | Chronic pulmonary heart disease | 8.17E-03 |
|  |  | Taurine and hypotaurine metabolism | 5.63E-02 | Cancer, suspected or other | 8.80E-03 |
|  |  | cGMP-PKG signaling pathway | 5.70E-02 | Carcinoma in situ of skin | 8.80E-03 |
| purple | 35 | Systemic lupus erythematosus | 4.48E-62 | Disorders of iron metabolism | 2.37E-11 |
|  |  | Alcoholism | 1.04E-57 | Symptoms of the muscles | 4.64E-05 |
|  |  | Viral carcinogenesis | 2.03E-23 | Adult failure to thrive | 3.61E-02 |
|  |  | Necroptosis | 1.12E-08 | Respiratory abnormalities | 4.96E-02 |
|  |  | Transcriptional misregulation in cancer | 2.90E-08 | Bursitis | 5.12E-02 |
| red | 1825 | Metabolism of xenobiotics by cytochrome P450 | 2.80E-04 | Genital prolapse | 6.45E-04 |
|  |  | Chemical carcinogenesis | 3.42E-04 | Breast cancer | 2.55E-03 |
|  |  | Neuroactive ligand-receptor interaction | 1.31E-03 | Osteoarthrosis, localized, primary | 2.73E-03 |
|  |  | Caffeine metabolism | 6.53E-03 | Heart failure with preserved EF [Diastolic heart failure] | 2.88E-03 |
|  |  | Protein digestion and absorption | 6.83E-03 | Other venous embolism and thrombosis | 4.09E-03 |
| yellow | 678 | Dilated cardiomyopathy (DCM) | 3.67E-03 | Cancer of stomach | 2.18E-03 |
|  |  | Adrenergic signaling in cardiomyocytes | 3.86E-03 | Pelvic peritoneal adhesions, female (postoperative) (postinfection) | 5.20E-03 |
|  |  | Cardiac muscle contraction | 4.83E-03 | Cholecystitis without cholelithiasis | 5.85E-03 |
|  |  | Glutamatergic synapse | 5.35E-03 | Cancer of eye | 8.57E-03 |
|  |  | Hypertrophic cardiomyopathy (HCM) | 8.07E-03 | Elevated cancer antigen 125 [CA 125] | 8.57E-03 |
